# Ambience-sensitive optical refraction in ferroelectric nanofilms of NaNbO_3_

**DOI:** 10.1088/1468-6996/15/4/045001

**Published:** 2014-07-02

**Authors:** Marina Tyunina, Dagmar Chvostova, Oliva Pacherova, Tomas Kocourek, Miroslav Jelinek, Lubomir Jastrabik, Alexander Dejneka

**Affiliations:** 1 Microelectronics and Materials Physics Laboratories, University of Oulu, PO Box 4500, FI-90014 Oulun yliopisto, Finland; 2 Institute of Physics, Academy of Sciences of the Czech Republic, Na Slovance 2, 182 21 Prague 8, Czech Republic

**Keywords:** epitaxial, nanofilm, refraction, NaNbO_3_

## Abstract

Optical index of refraction *n* is studied by spectroscopic ellipsometry in epitaxial nanofilms of NaNbO_3_ with thickness ∼10 nm grown on different single-crystal substrates. The index *n* in the transparency spectral range (*n* ≈ 2.1 – 2.2) exhibits a strong sensitivity to atmospheric-pressure gas ambience. The index *n* in air exceeds that in an oxygen ambience by *δn* ≈ 0.05 – 0.2. The thermo-optical behaviour *n*(*T*) indicates ferroelectric state in the nanofilms. The ambience-sensitive optical refraction is discussed in terms of fundamental connection between refraction and ferroelectric polarization in perovskites, screening of depolarizing field on surfaces of the nanofilms, and thermodynamically stable surface reconstructions of NaNbO_3_.

## 1. Introduction

Perovskite-structure *AB*O_3_ ferroelectric crystals have long been employed in optical and photonic devices owing to a number of remarkable properties including wide bandgaps (>3 eV), large electro-optical and nonlinear optical coefficients, high static dielectric constants, and the possibility of sustaining and switching a spontaneous polarization [1–3]. In the last decades, benefits of epitaxial thin films of ferroelectrics for applications in integrated optics have been demonstrated [4]. The large electro-optic coefficient of the ferroelectric films is the major advantage for applications.

The physical mechanisms responsible for the electro-optical effects in perovskite ferroelectrics have been analysed theoretically in seminal work [5] and more recent first-principles study [6]. The key factor is the presence of ferroelectric polarization, affecting the electron energy. There is a fundamental connection between the ferroelectric polarization and the optical index of refraction *n* in perovskite ferroelectrics. Importantly, the polarization in epitaxial ferroelectric films can differ from that in bulk samples leading to new optical properties of the films compared to crystals.

Compared to bulk ferroelectric crystals, mismatch between crystal structure, lattice parameters, and thermal expansion coefficients of the film and substrate materials can enable growth of new crystal phases and/or create lattice strain in heteroepitaxial ferroelectric films. As a consequence, the polarization changes [7–10], and the index *n* can change correspondingly in epitaxial films compared to crystals. Additionally, the ferroelectric polarization depends on the thickness of ferroelectric films. The intrinsic surface charge of the polar phase, or the polarization charge, creates an electric field opposing the ferroelectric polarization. The depolarizing field increases with decreasing films thickness and can destroy the ferroelectric polarization. However, either electronic charge in metallic electrodes or extra ions, or point defects at the electrode-less surfaces can provide screening of the depolarizing field and thus stabilize the ferroelectric phase in films with thickness of a few nanometres only, or nanofilms. Thus polarization can vary due to variation of ionic compensation on the surface of ferroelectric film.

Here we show that due to fundamental connection between polarization and optical properties of perovskite ferroelectrics, the sensitivity of ferroelectric polarization to surface state in ferroelectric nanofilms can lead to ambience-sensitive optical refraction. We report on atmospheric-pressure ambience-sensitive index of refraction in electrode-less epitaxial nanofilms of NaNbO_3_. Usually, bulk NaNbO_3_ is considered to be antiferroelectric at room temperature [11], although the coexistence of the antiferroelectric and ferroelectric phases has been detected too [12]. Here the ferroelectric phase of NaNbO_3_ is achieved using epitaxial growth on cubic single-crystal substrates [9, 13]. Profound changes of refraction are obtained at atmospheric pressure in the NaNbO_3_ nanofilms. The index *n* stabilized in air significantly exceeds the index in an oxygen ambience. This observation is discussed in terms of thermodynamically stable surface reconstructions of NaNbO_3_ resulting in different screening of the depolarizing field. We anticipate that ambience sensitivity of optical refraction may exist in other nanoscale perovskite ferroelectrics. Moreover, it may be possible to tune this sensitivity by selecting an appropriate perovskite composition. The phenomenon is important for advanced optical devices, chemical sensing, and catalysis.

## 2. Experiment

NaNbO_3_ films were grown by pulsed laser deposition onto (La_0.18_Sr_0.82_)(Al_0.59_Ta_0.41_)O_3_ (001), SrTiO_3_ (001), MgO (001), and DyScO_3_ (011) single-crystal substrates (LSAT, STO, MgO, and DSO for brevity) at elevated temperature *T*_PLD_ = 973 K in oxygen ambience. The pressure of optically clean oxygen was 20 Pa during deposition and it was raised to atmospheric pressure during post-deposition cooling or annealing. The thickness of the films was 9–14 nm as determined from the Laue satellites in x-ray diffraction (XRD) patterns and from ellipsometric data.

The room-temperature crystal structure of the grown NaNbO_3_ films was studied by XRD on high-resolution Bruker D8 DISCOVER DAVINCI and Bruker D8 DISCOVER SUPER SPEED SOLUTION diffractometers using Cu K*α* radiation. Reciprocal space mapping around the (033) and (303) reciprocal lattice points was performed using a rotating anode tube. The lattice parameters were refined from several Bragg diffractions using substrate diffractions as a reference. The measurements were carried out in atmospheric air. The XRD results are related to the air-stabilized samples discussed below.

The optical properties of the films were explored by variable-angle spectroscopic ellipsometry (VASE) on a J A Woollam ellipsometer [14, 15]. The ellipsometric data were collected over a spectral range from 0.74 to 9.0 eV and at different angles of incidence. The data analysis was based on the numerical inversion and was performed using the WVASE32 software package. The experimental ellipsometric spectra were fitted using a model considering a stack of semi-infinite substrate, film, surface-roughness layer, and ambient air. The parameterization of the initial dielectric functions of the films was performed using the multi-oscillator model. The optical properties of the surface-roughness layer were represented by a Bruggeman effective medium approximation. The temperature evolution of the index of refraction *n*, or thermo-optical behaviour, was studied at a photon energy of 2 eV in atmospheric air. The dielectric functions and the optical properties of the substrates were determined using separate independent measurements.

## 3. Results and discussion

### 3.1. Epitaxial films of NaNbO_3_

In its bulk form at *T*_PLD_, NaNbO_3_ has a cubic perovskite-type crystal structure with the lattice parameter *a*_0_ ≈ 3.937 Å.^5^ For a coherent cube-on-cube-type growth of NaNbO_3_ on top of the substrates employed here, the biaxial in-plane misfit strain *s*_*a*_ in NaNbO_3_ is expected to be *s*_*a*_ = (*a*_0_/*a*_*s*_ − 1), where *a*_*s*_ is the lattice parameter of a square mesh of the substrate surface. The in-plane strain at *T*_PLD_ would be large ∼10% on MgO and smaller (0–1%) on other substrates, suggesting possible pseudo-morphic growth of NaNbO_3_ on LSAT, STO, and DSO, and an abrupt misfit relaxation in the vicinity of MgO. The lattice strain can change upon cooling due to a mismatch between the thermal expansion coefficients of NaNbO_3_ and the substrate materials. The theoretical room-temperature in-plane strain is listed in table 1.

**Table 1.** The in-plane lattice parameters of the substrates (*a*_*s*_), the theoretical in-plane strain (*s*_*a*_), and the measured out-of-plane lattice parameters (*c*) and strain (*s*_*c*_) in the NaNbO_3_ films.

Substrate	*a*_*s*_ (Å)	*s*_*a*_ (%)	*c* (Å)	*s*_*c*_ (%)
LSAT (001)	3.868	−1.0	3.931	0.6
SrTiO_3_ (001)	3.905	−0.1	3.906	<0.1
MgO (001)	4.213	7.8	3.899	−0.2
DyScO_3_ (011)	3.943	0.9	3.863	−1.1

**Figure 1.**
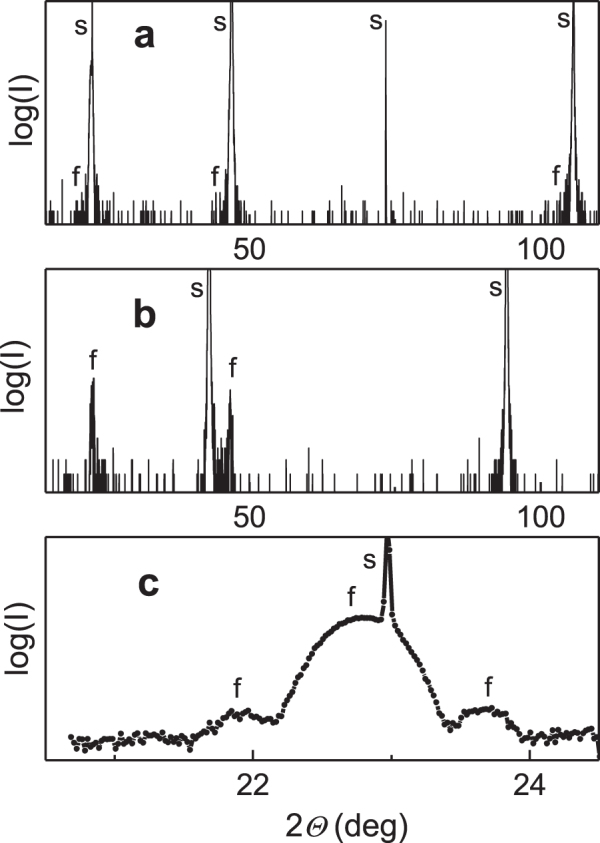
Typical *Θ*-2*Θ* x-ray diffraction patterns of the NaNbO_3_ nanofilms on (a) STO, (b) MgO, and (c) DSO substrates. The diffractions of the films and substrates are marked by *f* and *s*, respectively. The Laue satellites around perovskite (001) diffraction in (c) indicate high crystal perfection and smooth surface of the film.

The XRD studies revealed that all films are highly oriented, with (001) planes parallel to the (001) planes of LSAT, MgO, and STO substrates, and (011) plane of DSO (figure 1). Reciprocal space mapping showed a cube-on-cube type epitaxial relationship. The measured out-of-plane lattice parameters (table 1) are found to differ from those of the perovskite cell of NaNbO_3_ at room temperature. The crystal structure of the NaNbO_3_ films can be interpreted as pseudo-cubic (on STO) or metrically tetragonal (on other substrates). Compared to the perovskite cell of bulk NaNbO_3_, the measurements evidence the presence of anisotropic lattice strain in all films except in the NaNbO_3_ film on STO [13]. The strain is approximately 1% on LSAT (in-plane compression and out-of-plane elongation), 0.6% on DSO (in-plane expansion and out-of-plane compression), and it is weak ∼0.2% on MgO (in-plane expansion and out-of-plane compression). The strain is consistent with the theoretical considerations discussed above.

According to first-principles calculations, the deposited NaNbO_3_ films are expected to be in the ferroelectric *r*-state [9]. In terms of the perovskite unit cell of the (001) oriented pseudo-cubic or tetragonal NaNbO_3_ films, the in-plane component of polarization *P*_*a*_ lies along the in-plane [110] direction in the NaNbO_3_ film and the out-of-plane component of polarization *P*_*c*_ lies along the [001] direction, normal to the substrate surface. Notice that the *r*-phase can be treated as monoclinic phase, whose unit cell is different from the discussed perovskite unit cell. Importantly, the total polarization is [*P* = (*P*_*a*_^2^ + *P*_*c*_^2^)^1/2^] in the *r*-phase. The depolarizing field is related to the out-of-plane polarization *P*_*c*_ only.

### 3.2. Optical refraction

**Figure 2.**
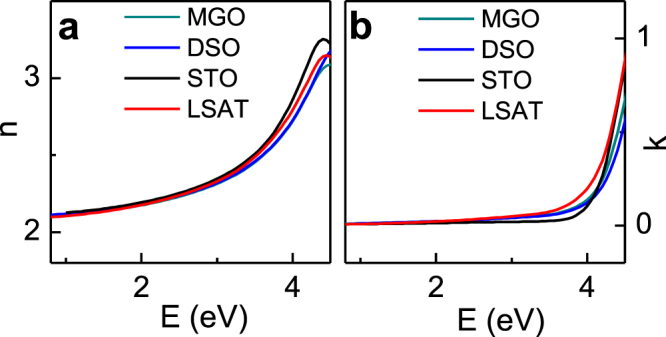
The room-temperature (a) index of refraction *n* and (b) extinction coefficient *k* as a function of photon energy *E* in the NaNbO_3_ films on different substrates.

The room-temperature optical constants of the NaNbO_3_ films were extracted from the ellipsometric data for the photon energies *E* = 0.8–9.0 eV. A detailed analysis of the spectra will be reported elsewhere. The lower energy fractions of the spectra are presented in figure 2. They indicate the main optical transition at *E* > 4.3 eV and show that the films are transparent at *E* < 3 eV. The index *n* was then studied at the photon energy *E* = 2 eV, which is in the transparency range.

**Figure 3.**
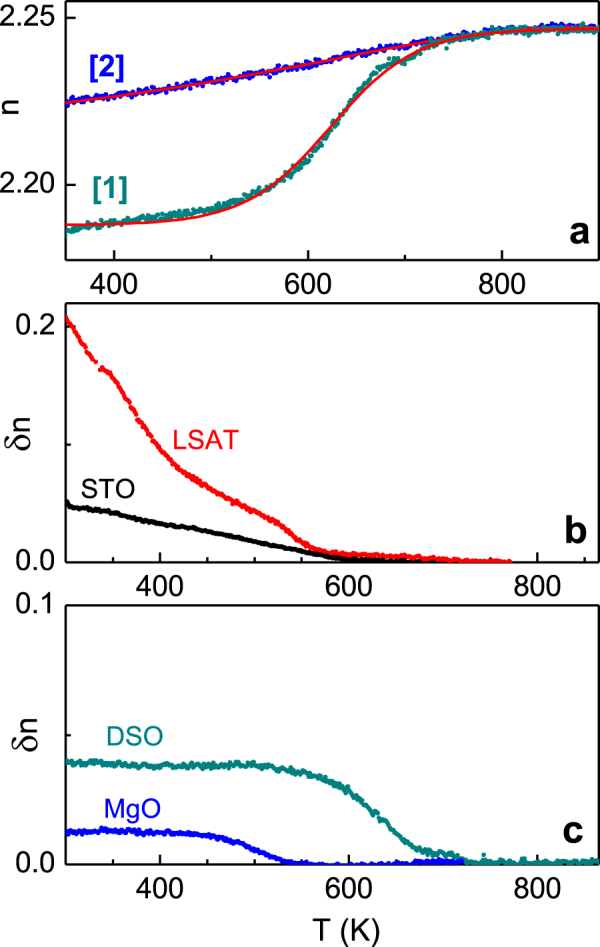
(a) The index of refraction *n* as a function of temperature *T* measured on heating of the as-deposited (curve 1) and the air-stabilized (curve 2) NaNbO_3_ film on DSO substrate. (b), (c) The difference *δn* between the curves (2) and (1) in the NaNbO_3_ films on different substrates.

The thermo-optical behaviour *n*(*T*) was investigated in atmospheric air. The films were subjected to heating, consequent cooling, and additional thermal cycling. The typical thermo-optical relaxation behaviour is shown in figure 3(a) for the NaNbO_3_ film on DSO. The index *n*(*T*) of the as-deposited film (curve (1)) is considerably smaller than that after heating in air (curve (2)) over a broad range of temperatures. The difference *δn*(*T*) between the thermo-optical characteristics is calculated by subtracting the data obtained in the as-deposited film (curve (1)) from those obtained after heating in air (curve (2)). The difference *δn*(*T*) is significant for all films (figures 3(b), (c)). The thermo-optical behaviour does not change upon further heating or cooling runs in air, i.e. the curves (2) are reproduced steadily during thermal cycling in air. Remarkably, the high-temperature annealing in oxygen of the air-stabilized films (723 K, 10^5^ Pa, 2–24 h) is found to result in recovery of the behaviour (1). Behaviour (2) is also achieved by maintaining the as-deposited films at room temperature in atmospheric air for several days, after which it remained unchanged for at least two years.

The sensitivity of the index *n* to gas ambience is very strong. Indeed, the detected variation *δn* of refraction index can be as huge as *δn* ≈ 0.2 (in the NaNbO_3_ film on LSAT). This value exceeds considerably refraction variations which are typically employed in electro-optic devices. The ambience-sensitive refraction can thus enable novel opto-chemical sensors. In order to get better insight in the phenomenon, the thermo-optical behaviour in the NaNbO_3_ films was further analysed.

### 3.3. Ferroelectric polarization

**Figure 4.**
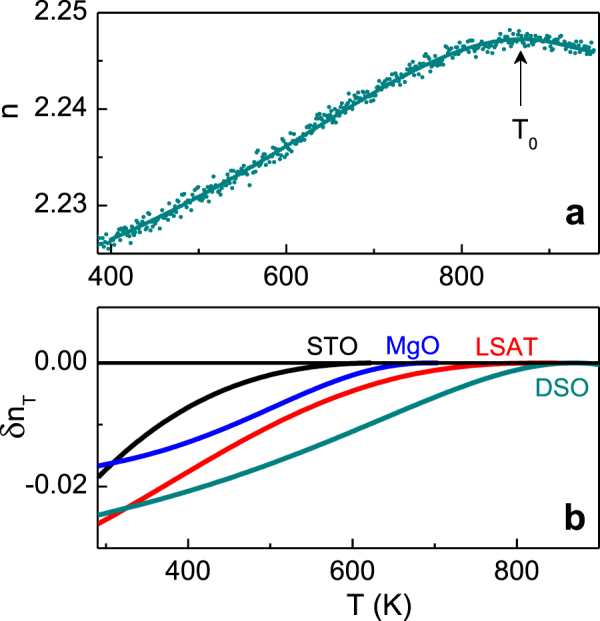
(a) The index of refraction *n* as a function of temperature *T* in the air-stabilized NaNbO_3_ film on DSO substrate. The arrow shows the temperature *T*_0_ of the para-to-ferroelectric phase transition. (b) The difference *δn*_*T*_ between the low-temperature index *n*(*T*) and the index *n*_0_(*T*_0_) in the air-stabilized NaNbO_3_ films on different substrates. In (b) the data are smoothed.

The thermo-optical behaviour evidences the presence of a phase transition at a certain temperature *T*_0_. The temperature *T*_0_ is determined here as that at which the sign of the derivative (d*n*/d*T*) changes (figure 4(a)). The sign is known to be negative in the high-temperature paraelectric state and positive in the low-temperature ferroelectric state of perovskite ferroelectrics [5, 6, 14]. The difference [*δn*_*T*_ = *n*(*T*) − *n*_0_(*T*_0_)] is found to be negative for temperatures below *T*_0_ in all films (figure 4(b)), confirming the low-temperature ferroelectric state in the NaNbO_3_ films. The observation of ferroelectric behaviour is consistent with the previous theoretical and experimental studies of epitaxial NaNbO_3_ films [9, 16, 17, 18].

**Figure 5.**
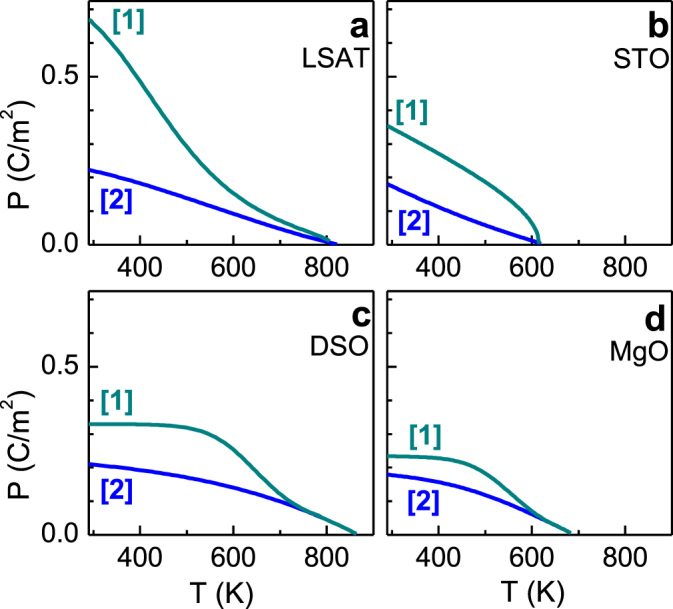
The average polarization *P* in the as-deposited (curves 1) and air-stabilized (curves 2) NaNbO_3_ films.

Compared to the index *n*_0_ in the PE state, the appearance of polarization *P* in the ferroelectric state leads to the index *n*, which can be related to the total polarization as follows [5]:

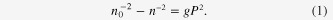
Here *g* is the quadratic electro-optic coefficient. Although the coefficient *g* is a tensor and is unknown for epitaxial NaNbO_3_, the ferroelectric polarization in the NaNbO_3_ films can be estimated using the average relationship (1) with *g* = 0.1 m^4^ C^−2^ [5]. The estimated average polarization *P* is plotted in figure 5. The polarization is larger in the oxygen-stabilized state (*P*_1_) than in the air-stabilized state (*P*_2_) in all films. The room-temperature oxygen-stabilized polarization *P*_1_ and the difference between *P*_1_ and *P*_2_ are the largest in the NaNbO_3_ film on LSAT. Importantly, the NaNbO_3_/LSAT film exhibits the strongest out-of-plane lattice elongation and, correspondingly, the largest out-of-plane polarization *P*_*c*_.

**Figure 6.**
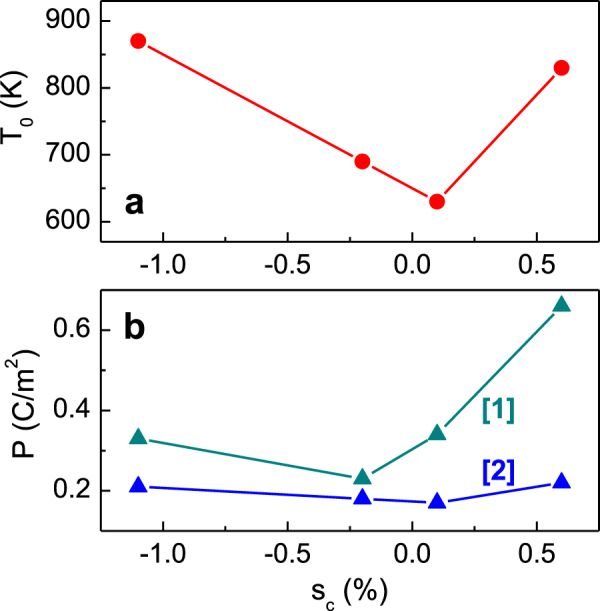
(a) The temperature *T*_0_ of the phase transition and (b) the room-temperature polarization *P* in the NaNbO_3_ films as a function of the measured out-of-plane strain *s*_*c*_. In (b) the oxygen-stabilized polarization (curve 1) and the air-stabilized polarization (curve 2) are shown.

A correlation between the measured out-of-plane strain *s*_c_ and the ferroelectric properties in the NaNbO_3_ films is seen from figure 6. The temperature *T*_0_ of the phase transition increases with increasing magnitude of strain. This behaviour of *T*_0_ agrees well with the strain–temperature phase diagrams typical for epitaxial ferroelectric films [7]. Also increase of polarization with increasing strain magnitude is consistent with theoretical calculations [8–10]. Considering the presence of ferroelectric state in epitaxial films of antiferroelectric NaNbO_3_, the sensitivity of polarization and optical refraction to gas ambience can be related to ambience-sensitive screening of the out-of-plane depolarizing field in the nanofilms of NaNbO_3_.

### 3.4. Surface reconstruction

In the absence of a bottom electrode layer in the studied NaNbO_3_ films, the ferroelectric polarization depends on the screening conditions both on the NaNbO_3_ surfaces and at the NaNbO_3_—substrate interfaces. Because the structure of the buried interfaces is unlikely to change in our experiments, the interfacial screening is assumed to be constant. The difference between the oxygen-stabilized and air-stabilized polarization can originate from different screening conditions on the NaNbO_3_ surfaces, which in turn are connected to different thermodynamically stable surface states of NaNbO_3_ achieved in oxygen and in air.

An equilibrium surface stoichiometry is generally determined by a minimum Gibbs free energy *G*, which can be presented for an *AB*O_3_ film as follows:

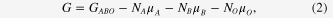
where *G*_*ABO*_ is the free energy of the film, *μ*_*A*_, *μ*_*B*_, and* μ*_*O*_ are the chemical potentials of the *A*-site and *B*-site atoms, and oxygen, respectively, and *N*_*A*_, *N*_*B*_, and *N*_*O*_ are the number of *A*, *B*, and O atoms in the surface layer [19, 20]. Because the chemical potentials are a function of temperature and pressure, numerous possible (*A*O)-type and (*B*O_2_)-type surface structures have been obtained theoretically for FEs such as BaTiO_3_ and PbTiO_3_ [19, 20]. However in contrast to PbTiO_3_, BaTiO_3_, and other *A* ^2+^*B* ^4+^O_3_ perovskites possessing charge-neutral atomic (*A* ^2+^O) and (*B* ^4+^O_2_) planes, the atomic planes (Na^1+^O) and (Nb^5+^O_2_) of NaNbO_3_ are charged. The charge-imbalanced (*A* ^1+^O)- or (*B* ^5+^O_2_)-terminated surfaces cannot be stable, and a surface termination with an admixture of (*A* ^1+^O)^−^ and (*B* ^5+^O_2_)^+^ layers has been suggested [21]. Although the surface phase diagram of NaNbO_3_ is not known and may be very complex, expression (2) is employed here for a qualitative explanation.

The free energy of the NaNbO_3_ film is assumed to be the same in air and in oxygen at atmospheric pressure. The chemical potentials *μ*_*Na*_, *μ*_*Nb*_, and *μ*_*O*_ of Na, Nb, and O, respectively, depend on the partial oxygen pressure. Compared to an oxygen ambience (oxygen pressure of 10^5^ Pa), a lower partial oxygen pressure in atmospheric air (∼0.2 × 10^5^ Pa) leads to changes in the chemical potentials and, hence, in the numbers *N*_Na_, *N*_Nb_, and *N*_O_ of Na, Nb, and O atoms at the surface. Moreover, the presence of nitrogen and water vapour can cause additional changes in the chemical potentials in air and can affect the surface composition. Although knowledge of the NaNbO_3_ surface reconstructions requires a first-principles analysis, it is qualitatively clear that the air-stabilized and the oxygen-stabilized NaNbO_3_ films may have different surface compositions leading to different screening of the depolarizing field. This effect is manifested in large variations of the polarization and the index of refraction in the epitaxial NaNbO_3_ nanofilms.

It is worth mentioning that ambience-induced change of polarization has been previously detected using synchrotron-based studies of epitaxial PbTiO_3_ nanofilm with bottom electrode [22, 23]. The chemical control of polarization in the PbTiO_3_ nanofilm has been obtained at very low gas pressures varying from the ultra-high vacuum conditions (10^−5^ Pa) to the low-pressure oxygen ambience (10^3^ Pa). The present work shows atmospheric-pressure ambience-sensitive ferroelectric polarization and optical refraction in nanofilms without electrodes. The ambience-sensitive optical refraction may exist in other nanoscale perovskite ferroelectrics and it may be tuned by selecting an appropriate perovskite composition.

## 4. Conclusions

The optical index of refraction *n* is studied by spectroscopic ellipsometry in electrode-less cube-on-cube-type epitaxial NaNbO_3_ films with thicknesses of 9–14 nm and anisotropic lattice strain of (0–1)%. The room-temperature index *n* in the transparency spectral range is approximately 2.1–2.2 and it exhibits a strong sensitivity to atmospheric-pressure gas ambience. The index *n* in air significantly exceeds that in an oxygen ambience, with the difference as large as *δn* = 0.2.

The thermo-optical behaviour *n*(*T*) indicates ferroelectric state in the NaNbO_3_ nanofilms, with the strain-dependent polarization and temperature of phase transition. The observed ambience-sensitive optical refraction is explained using the fundamental connection between refraction and ferroelectric polarization in perovskites, and considering screening of the depolarizing field on ambience-sensitive thermodynamically stable surfaces of the NaNbO_3_ nanofilms.
